# Activation of Opioid Receptors Attenuates Ischemia/Reperfusion Injury in Skeletal Muscle Induced by Tourniquet Placement

**DOI:** 10.1155/2021/6699499

**Published:** 2021-01-15

**Authors:** Yue-Xian Guo, Gui-Ying Wang, Wen-jie Cheng, Cai-Zhen Yan, Shuang Zhao, Zhao Li, Peng Liu, Xiu-Li Wang

**Affiliations:** ^1^Department of Surgery, Hebei Medical University Third Affiliated Hospital, Shijiazhuang, Hebei 050051, China; ^2^Department of Anesthesiology, Hebei Medical University Third Affiliated Hospital, Shijiazhuang, Hebei 050051, China; ^3^Department of Pharmacology, Hebei Medical University, Shijiazhuang, China

## Abstract

**Method:**

Mice were randomly assigned to the sham, I/R, Oxy, and I/R with Oxy groups. Oxy was injected intraperitoneally 30 min before tourniquet placement. Morphological changes of the gastrocnemius muscle in these mice were assessed by hematoxylin-eosin (HE) staining and electron microscopy. Expression levels of TLR4, NF-*κ*B, SIRT1, and PGC-1*α* in the skeletal muscles were detected by western blot. Blood TNF-*α* levels, gastrocnemius muscle contractile force, and ATP concentration were examined.

**Results:**

Compared with the I/R group, Oxy pretreatment attenuated skeletal muscle damage, decreased serum TNF-*α* levels, and inhibited the expression levels of TLR4/NF-*κ*B in the gastrocnemius muscle. Furthermore, Oxy treatment significantly increased serum ATP levels and the contractility of the skeletal muscles. SIRT1 and PGC-1*α* levels were significantly reduced in gastrocnemius muscle after I/R. Oxy pretreatment recovered these protein expression levels.

**Conclusion:**

Tourniquet-induced acute limb I/R results in morphological and functional impairment in skeletal muscle. Pretreatment with Oxy attenuates skeletal muscle from acute I/R injury through inhibition of TLR4/NF-*κ*B-dependent inflammatory response and protects SIRT1/PGC-1*α*-dependent mitochondrial function.

## 1. Introduction

Tourniquet placement is a universal approach used to restrict blood flow to the operating fields during vascular and orthopedic surgery [1]. However, tourniquet placement leads to a significant acute limb ischemia/reperfusion (I/R) injury, characterized by inflammation, tissue edema, muscle necrosis, and microvascular perfusion deficits [2]. Furthermore, skeletal muscle injury may continue even after the restoration of blood flow [2, 3]. These complications have limited the usage of tourniquet [4]. Although tourniquet-induced I/R injury has been recognized, the pathophysiologic mechanisms need to be clarified.

Although tourniquets are an essential approach used in patients who receive vascular or orthopedic surgeries, there are several regional and systemic complications associated with utilizing this device. These complications include hyperdynamic response, peripheral nerve block, and skeletal muscle damage [5, 6]. Prolonged limb tourniquet placement and the subsequent restoration of blood flow after cessation of tourniquet placement result in inflammation related to ischemia in skeletal muscle. Moreover, inflammation responses are often amplificated and lead to a complex cytokine cascade that is associated with secondary remote organ damage [7]. Therefore, exploring effective strategies to minimize the damage of tourniquet-induced acute limb I/R injury significantly improves outcomes and quality of life for patients who have utilized this device.

Acute limb I/R causes skeletal muscle damage due to several mechanisms [8]. Skeletal muscle is characterized by its high content of mitochondria, which has a lower threshold for ischemic damage than other tissues such as nerve or bone. Increasing evidence supports the notion that the sirtuin family of deacetylases (SIRTs) is one of the key regulators capable of promoting mitochondriogenesis and enhancing respiration chain reaction in mitochondria [9]. SIRT1 can coordinate with peroxisome proliferator-activated receptor *γ* coactivator-1*α* (PGC-1*α*) to promote metabolic adaptation and enhance mitochondriogenesis [10, 11]. Toll-like receptors (TLRs) are ubiquitous to activate the innate immune system in response to pathogens and stressors [12]. Among TLR subtypes, TLR4 leads to activation of nuclear factor kappa B (NF-*κ*B) to trigger the expression of many proinflammatory genes such as IL-1 and TNF-*α* [13–15].

Oxycodone (Oxy), a *μ* and *κ* opioid receptor agonist, is increasingly used worldwide to treat various painful disorders [16]. Recently, several studies have suggested that Oxy not only provides potent analgesia but also inhibits the inflammatory response [17, 18]. To date, the effects of Oxy on TLR4-related inflammation responses during tourniquet-induced I/R injury have not been evaluated. In this study, we hypothesized that Oxy exerts protection against tourniquet-induced acute I/R injury. We also explored the underlying mechanism in a mouse model of acute hind limb I/R.

## 2. Materials and Methods

### 2.1. Experimental Animals and Procedures: Tourniquet-Induced Acute Hind Limb I/R Injury

This study was carried out by using C57BL6 male mice (10-12 weeks old). The experimental procedures were approved by the Animal Care and Use Committee of the Hebei Medical University and performed in accordance with the National Institutes of Health guidelines for the Care and Use of Laboratory Animals. Ethical approval for this study (ethical protocol number 2019-0183) was provided by the Ethical Committee of the Third Hospital of Hebei Medical University (Chairperson Dr. Yong Shen) on 9 January 2019.

The C57BL6 male mice were subjected to 3 h ischemia in a unilateral hind limb by tourniquet and followed by 24 h reperfusion (I/R), as described previously [1]. In brief, acute hind limb ischemia was induced by applying a calibrated orthodontic rubber band as a tourniquet to the left proximal hind limb. The reperfusion was initiated by the removal of the rubber band. A heating pad was used to maintain body temperature during the procedure. The degree of ischemia/reperfusion (I/R) injury induced by the tourniquet was assessed by blood flow to the hind limb measured by a microcirculation imaging system. Mice were assigned to the sham group, the I/R group, and the Oxy group randomly. The mice were anesthetized by sodium pentobarbital (80.0 mg/kg, intraperitoneal injection) before and during application of a calibrated orthodontic rubber band to the left proximal hind limb, rather than the whole period of 3 h ischemia and 24 h reperfusion. In addition, the mice were anesthetized by isoflurane (2% in pure O_2_ inhalation) when the images were taken. The mice in the I/R or Oxy group received a normal saline solution or 0.6 mg/kg Oxy (diluted in 0.9% NaCl, intraperitoneal injection) 30 min prior to application of the orthodontic rubber band, respectively. The mouse in the sham group was not applied an orthodontic rubber band. For harvesting tissue samples, the mice were euthanized by cervical decapitation under anesthesia with sodium pentobarbital (80.0 mg/kg, intraperitoneal injection).

### 2.2. Perfusion Imaging

We used a real-time microcirculation imaging system to determine limb blood flow. In brief, mice were anesthetized with isoflurane (2% in pure O_2_) and placed on a dark scanning surface. Blood flow of the dorsal surface of the right and left hind limbs was measured by a blood perfusion imager (PeriCam PSI System, Sweden), which is based on laser speckle contrast analysis technology. We performed tourniquet placement on the left limb, and the blood flow images were acquired before the ischemia procedure, during the ischemia procedure, and 24 h after reperfusion. These images of the mouse limbs were analyzed using PIMSoft (PeriCam PSI System). The left ischemic limb values were then normalized to that of the right (nonischemic) limb to calculate the percentage of blood perfusion for each mouse.

### 2.3. Evaluation of Skeletal Muscle Damage

Gastrocnemius muscles were obtained from mice in the sham group, the I/R group, and the Oxy group and fixed in 4% buffered paraformaldehyde followed by paraffin embedding. Then, the muscles were cut into longitudinal and transverse sections at the midpoints, then deparaffinized, hydrated, and stained with hematoxylin-eosin staining. Moreover, 2 *μ*m thick acrylic embedded cross and lengthwise sections were stained with Masson trichrome as described previously [19]. Stained sections were captured by a bright-field microscope equipped with a digital camera. A pathologist assessed tissue edema, hemorrhage, neutrophil infiltration, and myocyte damage in a blinded manner.

### 2.4. Electron Microscope (EM) Analysis

We observed the ultrastructure of the gastrocnemius muscles by EM analysis. The skeletal muscle tissues were removed from mice in each group and sectioned into 1-3 mm pieces and processed according to the method described in a previous study [20]. Sections were stained with uranyl acetate and lead citrate (Sigma-Aldrich, St. Louis, MO, USA), then observed by an H-7500/STEM transmission electron microscope (Hitachi Inc., Tokyo, Japan) at 15,000 magnification.

### 2.5. Measurement of TNF-*α* Level

In a separate experiment, after 24 h of reperfusion, mice in the sham group, the I/R group, and the Oxy group were euthanized by cervical decapitation under anesthesia with sodium pentobarbital (80.0 mg/kg, intraperitoneal injection), and blood samples were collected. The serum was separated by centrifugation and stored at -80°C for further analysis. TNF-*α* concentration was measured by an Enzyme-Linked Immunosorbent Assay (ELISA) Kit (ABclonal Biotechnology Co., Ltd., No.: 9680016107) according to the manufacturer′s instruction.

### 2.6. Measurement of ATP Concentration in Gastrocnemius Muscle

The gastrocnemius muscle tissues obtained from mice in the sham group, the I/R group, and the Oxy group were harvested and fixed at -80°C immediately. Then, 30.0 mg skeletal muscle samples were homogenized on ice with a perchloric acid solution in a tissue homogenizer (MP, FastPrep-24 5G). A 1.0 ml supernatant was taken to adjust the pH with a 0.5 M K_2_HPO_4_ solution after centrifugation at 5000 rpm for 10 min. According to the manufacturer's instruction, the concentration of ATP in gastrocnemius muscle was examined in duplication by a commercially available assay kit (Jiancheng Co., Nanjing, China, No.: A095).

### 2.7. Measurement of Gastrocnemius Muscle Contractile Force

We assessed the gastrocnemius muscle contractile force in the sham, I/R, and Oxy groups. Under anesthesia with pentobarbital sodium (80 mg/kg, i.p.), the left gastrocnemius muscles were quickly removed and rinsed in ice-cold modified Krebs-Henseleit (K-H) solution saturated by 95% O_2_ and 5% CO_2_. The K-H solution contained (in mmol/L) NaCl 118, KCl 4.7, CaCl_2_ 1.8, MgSO_4_·7H_2_O 1.2, KH_2_PO_4_ 1.2, NaHCO_3_ 25, glucose 11, and Hepes 10 (pH 7.4 ± 0.05, gassed with 95% O_2_ and 5% CO_2_). The gastrocnemius muscle's proximal end was fixed, while the distal tendon was attached to a mechanical force transducer (AD Instruments, Australia). An initial tension of 1 g was applied to the muscle preparations. The stimuli were delivered through a bipolar electrode placed in a chamber and connected to a stimulator. The gastrocnemius muscle was continuously perfused with K-H solution at 15 ml/min at 37°C for at least 1 h before the experiments. Individual twitch contraction of the gastrocnemius muscle was induced by the stimulation (5 V, 1 Hz, 1 ms pulse), which was repeated 30 times to induce tetanic contractile force (5 V, 120 Hz, 1 ms pulse). The PowerLab (AD Instruments) data acquisition system with LabChart7 software was used to record and analyze the muscle contraction.

### 2.8. Western Blotting Analysis of Protein Expression of TLR4, NF-*κ*B, SIRT1, and PGC-1*α* in Gastrocnemius Muscle

After 24 h reperfusion, gastrocnemius muscles from mice in the sham, I/R, and Oxy groups were rapidly taken and stored at -80°C until analysis. The muscle tissues were dissected and treated with RIPA Lysis Buffer (Santa Cruz, Dallas, TX, USA) for homogenization. The homogenates were centrifuged at 12,000 g for 20 min at 4°C to obtain total protein in the centrifuged supernatants. A bicinchoninic acid protein assay kit (Thermo Fisher Scientific, Waltham, MA) was used to determine protein concentration. Loading buffer at the same volume was added into the protein samples and was mixed and boiled for 10 min at 95°C, then separated using 10% sodium dodecyl sulfate-polyacrylamide gel electrophoresis (SDS-PAGE). Proteins in these samples were transferred onto a polyvinyldifluoride (PVDF) membrane at 200 mA for 3 h, and then, the membrane was blocked with 5% nonfat milk for 1 h. Membranes were cut into strips according to molecular weight and probed with rabbit anti-TLR4 (Abcam, Cambridge, MA), rabbit anti-NF-*κ*B antibody (Abcam, Cambridge, MA), rabbit anti-SIRT1 antibody (Abcam, Cambridge, MA), and rabbit anti-PGC-1*α* antibody (Abcam, Cambridge, MA) overnight at 4°C. After being washed by tris buffered saline with Tween (TBST), the membranes were incubated with HRP-conjugated goat anti-rabbit IgG (Thermo Fisher Scientific, Waltham, MA) for 2 h at room temperature. *β*-Actin (Cell Signaling Technology, Beverly, MA) was used as a control. Finally, the PVDF membrane was rewashed with TBST three times for 15 min each. Specific immunoreactivity was visualized by an enhanced chemiluminescence substrate (Thermo Fisher Scientific, Waltham, MA), and the specific bands were scanned for analysis using ImageJ.

### 2.9. Statistical Analysis

Statistical analysis was performed using SPSS17.0 (SPSS Inc., IL, USA). Data were expressed as the mean ± standard error of means (SEM). Normal distribution of data was confirmed with the Kolmogorov-Smirnov test and equal variance with Levene's test. For multigroup comparison, one-way analysis of variance (ANOVA) with the Bonferroni post hoc test was used to determine statistical significance. Statistical significance was considered *P* < 0.05.

## 3. Results

### 3.1. Oxy Treatment Increased Blood Flow after Tourniquet Placement on the Hind Limb

**Figure 1:**
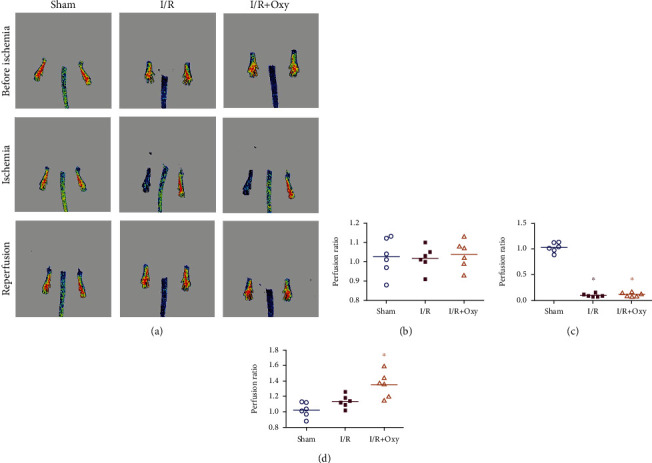
Oxy treatment increased blood flow after tourniquet placement on the hind limb. (a) Representative images of blood perfusion in the mouse hind limb obtained using laser speckle contrast imaging. The red color on the image represents high blood flow to the limb while the blue color represents low blood flow. Quantification of perfusion in the ischemic hind limb before ischemia (b), during ischemia (c), and during reperfusion for 24 h (d). I/R: ischemia-reperfusion; Oxy: oxycodone. Data are expressed as the mean ± SEM (*n* = 6 in each group); ^∗^*P* < 0.05 vs. sham.

We measured the blood flow by laser Doppler imaging and found that the blood flow did not differ between the left and right hind limbs before ischemia in all three groups of mice (*P* > 0.05, Figure 1). After the tourniquet was placed on the left limb, the blood flow in the I/R and I/R plus Oxy mice dropped to about 10.2% and 11.8% of the basal levels, respectively (*P* > 0.05, Figures 1(b)–1(d)). After reperfusion for 24 h, laser Doppler imaging demonstrated that the blood flow of mice in the I/R plus Oxy group significantly increased compared with that in the sham group (*P* < 0.05). The red color on the image represents high blood flow to the limb, while the blue color represents low blood flow. However, no difference was found between the I/R and Oxy groups (*P* > 0.05). This data indicates that Oxy preconditioning treatment increased blood flow of the hind limb during I/R.

### 3.2. Morphology Changes of Gastrocnemius Muscle in response to I/R Induced by Tourniquet Placement

**Figure 2:**
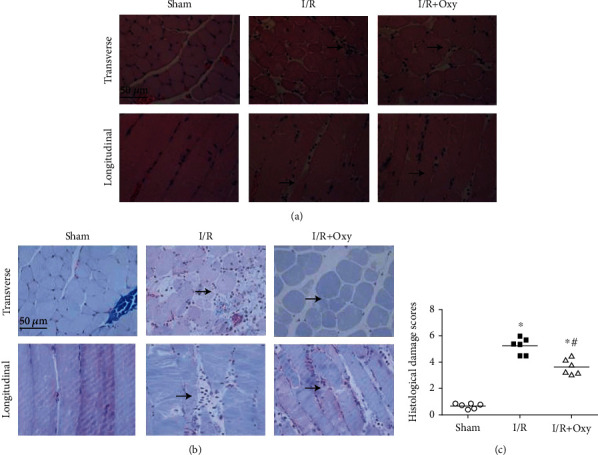
Morphology changes in gastrocnemius muscle tissues in response to tourniquet placement. (a) Representative histology in longitudinal and transverse sections of gastrocnemius muscles stained with hematoxylin-eosin. (b) Representative histology in cross and lengthwise sections of gastrocnemius muscles stained with Masson's trichrome. Arrows indicate the site of muscle injury. (c) Summary data of histology damage scores in sham, I/R, and I/R plus Oxy groups. I/R: ischemia-reperfusion; Oxy: oxycodone (*n* = 6 in each group).

We performed HE staining and Masson trichrome staining on gastrocnemius muscle to further determine the effect of tourniquet placement on the morphology changes of gastrocnemius muscle. The mice in the sham group exhibited normal muscle morphology, while the mice placed in tourniquet placement displayed disordered myofilament, swollen intercellular spaces, and ruptured sarcomere (Figure 2). Furthermore, we found that many leukocytes were infiltrated from the blood vessels into muscle tissue during tourniquet placement. In the tourniquet placement group treated with Oxy (0.6 mg/kg), the gastrocnemius muscle injury was significantly attenuated compared with that in the tourniquet placement group.

**Figure 3:**
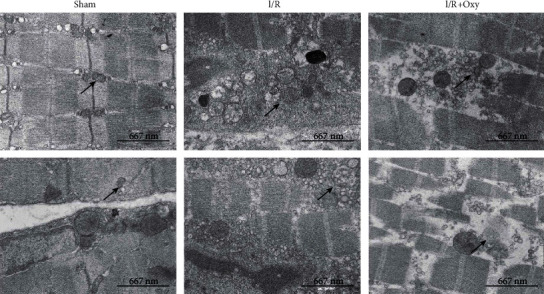
Electron microscope images of the gastrocnemius muscle tissues from all experimental groups. The scale was 667 nm at 15,000 magnification. I/R: ischemia-reperfusion; Oxy: oxycodone (*n* = 6 in each group).

In addition, we determined the effect of I/R induced by tourniquet placement on subcellular structure changes of gastrocnemius muscle cells by using EM imaging. The sham group displayed normal muscle cell morphology. However, disordered myofilament and swollen mitochondria with disrupted cristae were observed in the muscle tissue of mice subjected to tourniquet placement. In addition, gastrocnemius muscle cells displayed swollen sarcoplasmic reticulum in these mice (Figure 3). Oxy treatment (0.6 mg/kg) attenuated disruption in the intercellular matrix, mitochondria, and sarcoplasmic reticulum (Figure 3), suggesting that treatment with Oxy reduced muscle tissue injury induced by tourniquet placement.

### 3.3. Oxy Treatment Attenuated the Alteration of TNF-*α* and ATP Concentrations Induced by Tourniquet Placement

**Figure 4:**
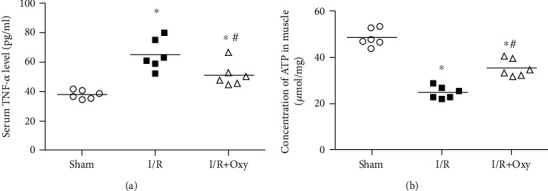
Oxy treatment attenuated alteration of TNF-*α* and ATP concentration induced by tourniquet placement. (a) Serum TNF-*α* level. (b) Concentration of ATP in gastrocnemius muscles. I/R: ischemia-reperfusion (*n* = 6 in each group); ^∗^*P* < 0.05 vs. sham; ^#^*P* < 0.05 vs. I/R. Oxy: oxycodone.

Tourniquet placement significantly increased TNF-*α* levels in serum compared with sham mice (*P* < 0.05). In Oxy-treated mice, this reduction of the serum TNF-*α* level was significantly attenuated in mice with tourniquet placement (Figure 4(a), *P* < 0.05). Furthermore, tourniquet placement significantly decreased ATP concentration in the gastrocnemius muscle compared with mice in the sham group. Treatment with Oxy significantly attenuated the reduction of ATP concentration induced by tourniquet placement (*P* < 0.05).

### 3.4. Oxy Treatment Attenuated the Impairment of Contractility of Gastrocnemius Muscles during Tourniquet Placement

**Figure 5:**
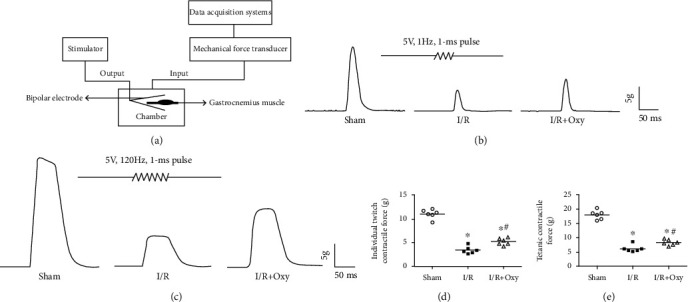
Oxy attenuated impairment of contractility of gastrocnemius muscles induced by a tourniquet. (a) Schematic diagram of experimental setup. (b) Individual twitch contraction of the gastrocnemius muscle. (c) Tetanic contractile force. (d, e) Quantitative analysis of individual twitch contractile force and tetanic contractile force in different groups. I/R: ischemia-reperfusion; Oxy: oxycodone. Data are expressed as the mean ± SE (*n* = 6 in each group); ^∗^*P* < 0.05 vs. sham; ^#^*P* < 0.05 vs. I/R.

After 3 h of ischemia induced by tourniquet placement and 24 h of reperfusion, the contractility of the gastrocnemius muscle in the I/R group was significantly lower than that of the sham mice (*P* < 0.05, Figure 5). Pretreatment with Oxy significantly attenuated this reduction of gastrocnemius muscle contractility in mice subjected to tourniquet placement (Figure 5). These data suggest that Oxy improves contractility of gastrocnemius muscles against injury during I/R.

### 3.5. Oxy Treatment Attenuated the TLR4/NF-*κ*B-Mediated Inflammation and SIRT1/PGC-1*α*-Dependent Mitochondrial Damage Induced during Tourniquet Placement

**Figure 6:**
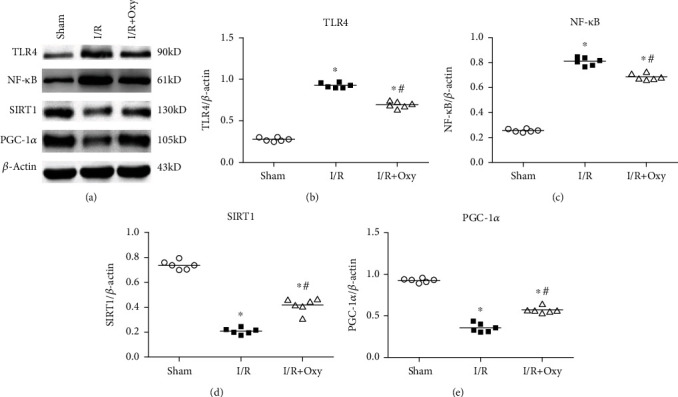
Oxy treatment decreased TLR4 and NF-*κ*B expression levels while increasing SIRT1 and PGC-1*α* levels in tourniquet-induced I/R in gastrocnemius muscle tissues. (a) Representative band images for TLR4, NF-*κ*B, SIRT1, and PGC-1*α* in the I/R and Oxy-treated I/R groups. (b–e) Summary data showing expression of TLR4 (b), NF-*κ*B (c), SIRT1 (d), and PGC-1*α* (e). I/R: ischemia-reperfusion; Oxy: oxycodone. Data are expressed as the mean ± SE (*n* = 6 in each group); ^∗^*P* < 0.05 vs. sham; ^#^*P* < 0.05 vs. I/R.

TLRs/NF-*κ*B signals are critical in triggering the release of many proinflammatory factors such as IL-1 and TNF-*α* [13–15]. To investigate if the TLRs/NF-*κ*B pathway is involved in gastrocnemius muscle injury during I/R induced by tourniquet placement, we examined the protein level of TLR4 and NF-*κ*B by western blot (Figure 6). Expression levels of TLR4 and NF-*κ*B were low in the sham group. However, after 3 h of tourniquet-induced ischemia and 24 h of reperfusion, these proteins were significantly elevated in gastrocnemius muscle tissues (*P* < 0.05). Oxy pretreatment significantly decreased TLR4 and NF-*κ*B expression levels compared with the I/R group (*P* < 0.05). These data suggest that Oxy attenuates TLR4/NF-*κ*B-mediated information response induced by tourniquet placement.

SIRT1/PGC-1*α* promotes metabolic adaptation and enhances mitochondriogenesis [10, 11]. Thus, we next determined the expression levels of SIRT1 and PGC-1*α* in gastrocnemius muscle tissues to test the hypothesis that pretreatment with Oxy increases mitochondrial function to attenuate the functional impairment of the skeletal muscle induced by tourniquet placement (Figure 6). We found that after I/R induced by tourniquet placement, SIRT1 and PGC-1*α* levels were significantly reduced in gastrocnemius muscle tissues (*P* < 0.05). Oxy pretreatment induced a profound increase in these proteins compared to the I/R group (*P* < 0.05).

## 4. Discussion

In the present study, we used a skeletal muscle I/R injury mouse model induced by 3 h of ischemia and 24 h of reperfusion. This model mimics the I/R injuries induced by tourniquet placement in patients who receive tourniquet placement during their postsurgery care. In this model, I/R induced functional and morphological damage in skeletal muscles. Oxy pretreatment significantly attenuated the I/R-induced functional and morphologic damage and inflammatory response in gastrocnemius muscle.

The hypoperfused blood flow—described as the no-reperfusion phenomenon—plays a critical role in skeletal muscle injury following I/R. Many mechanisms are proposed to be involved in developing the no-reperfusion phenomenon, which is characterized as interstitial edema formation and microvascular spasm [21]. Previous studies have shown that after cessation of 3 h ischemia, the perfusion rate gradually recovered over time. For example, the blood flow recovers to the level that is about 30-40% of the baseline level during the first 4 h of reperfusion and recovers to the level of about 80-90% of the baseline level after 24 h of reperfusion [22–24]. In this study, after 3 h of ischemia and 24 h of reperfusion, the perfusion recovered to about 95% of the baseline level, which was consistent with that reported previously. Moreover, we found that Oxy pretreatment increased the perfusion rate in gastrocnemius muscle after I/R induced by tourniquet placement.

Oxycodone, a semisynthetic opioid analgesic which is derived from naturally alkaloid thebaine, has been used to treat acute and chronic pain since 1917 and is increasingly used worldwide [25]. Being compared with other *μ*-opioid analgesics, Oxy provides potent analgesia with fewer side effects such as pruritus, low tolerance, and respiratory depression. Furthermore, several studies suggested that Oxy can relieve the inflammatory response [17, 18]. Thus, it is possible that Oxy treatment reduces the severity of skeletal muscle damage through an anti-inflammatory mechanism to preserve skeletal muscle function [8].

To test this hypothesis, we mimicked the critical limb syndrome in clinic induced by tourniquet placement in a mouse model. Findings from EM and histological analysis indicated that I/R induced by tourniquet placement led not only to morphological damage but also to leukocyte infiltration to skeletal muscle. Oxy treatment blunted gastrocnemius muscle injury induced by I/R and decreased inflammatory cell infiltration. TNF-*α*, as a sensitive inflammation factor, plays a critical role in initiating the inflammation of the innate immune system [26]. In this study, we demonstrated that acute limb I/R induced by tourniquet placement led to a systemic inflammatory response, which was characterized as an increase of the serum TNF-*α* level. Furthermore, we found that Oxy treatment significantly decreased the TNF-*α* concentration in the gastrocnemius muscle experiencing I/R induced by tourniquet placement. These data suggest that Oxy treatment blunted inflammatory responses related to the I/R injury induced by tourniquet placement.

As a primary component of the innate immune system, TLRs are an essential component in response to pathogens, stressors, and various cytokines. The activation of TLRs could trigger the release of a series of inflammatory factors including TNF-*α* [12, 13]. Previous studies have confirmed that activation of TLR4 could stimulate NF-*κ*B to lead to the transcription of many proinflammatory genes [27, 28]. However, the links between TLR4-related inflammation responses and skeletal muscle during I/R injury are identified. In our study, we examined the protein expression level of TLR4 and NF-*κ*B in skeletal muscle to explore TLR4-mediated mechanisms underlying the pretreatment with Oxy. Consistent with a previous study [15], we found that the expression level of TLR4 and NF-*κ*B was significantly higher in the I/R group than in the sham group. These data suggest that activating the TLR4/NF-*κ*B pathway plays a vital role in the pathophysiology of skeletal muscle I/R injury. Furthermore, we found that the levels of TLR4 and NF-*κ*B in skeletal muscle with I/R were significantly lowered in mice with I/R and treated by Oxy. Our findings provide evidence that Oxy pretreatment suppressed inflammation in muscle tissues experiencing I/R through inhibiting the activity of TLR4 and NF-*κ*B. Although opioid receptor agonists, including Oxy, produce significant increases in TLR4 signals in cultured HEK293 cells with stable expression of TL4 receptors [29], opioid receptor agonists inhibit LPS-induced TLR4 signals in HEK293 cells and in mice [30, 31]. We found that Oxy inhibited the activity of TLR4 in muscle tissues experiencing I/R. Thus, the mechanisms underlying Oxy-induced inhibition of TLR4 during I/R warrant further studies. In addition, other mechanisms, including oxidative stress and nitric oxide (NO) signaling, are significantly involved in the I/R injury in skeletal muscle and cardiac myocytes [32, 33]. Future studies are needed to clarify the role of oxidative stress and NO signaling in the protective effect of Oxy treatment on I/R injury induced by tourniquet placement.

To our knowledge, few studies investigated the effects of Oxy on energy metabolism in skeletal muscle during acute limb I/R injury. Thus, we made our efforts to explore the possible mechanisms involved in energy metabolism during skeletal muscle I/R injury. In our study, the skeletal muscle tissues in Oxy group mice have higher ATP concentration and contractile force compared with the I/R group. These data strongly support the notion that Oxy can improve mitochondrial function and prevent muscle damage during I/R. This finding suggests that Oxy suppressed the inflammatory response and reduced the functional impairment of the skeletal muscle.

The ATP generation from mitochondria is crucial for determining mitochondrial content and function. Accumulating evidence demonstrated that mitochondrial dysfunction is significantly involved in the pathogenesis of many inflammation-associated diseases [34]. Thus, drugs and nutrients that improve mitochondrial function could be beneficial in preventing those relevant diseases [35]. Peroxisome proliferator-activated receptor *γ* coactivator-1*α* (PGC-1*α*) critically regulates mitochondrial biogenesis through controlling the expression of mitochondrial DNA-encoded intramitochondrial proteins and modulate mitochondrial dynamics [36]. Another group of key regulators capable of promoting mitochondrial biogenesis is SIRTs [9]. Among them, SIRT1 is involved in inflammation and metabolic diseases [37, 38]. In particular, SIRT1's function is coordinated with PGC-1*α* to promote metabolic adaptation and enhance mitochondrial biogenesis [10, 11]. In this study, we found that mitochondrial function, represented by ATP levels and contractile force, was improved by Oxy treatment in skeletal tissues experiencing acute limb I/R injury. These findings led us to speculate that Oxy treatment may improve mitochondrial function such as mitochondrial biogenesis through activating the SIRT1/PGC-1*α* signaling pathway. As expected, we found that SIRT1 and PGC-1*α* levels were significantly reduced in gastrocnemius muscle tissues after I/R. Furthermore, Oxy pretreatment significantly increased these proteins' expression in gastrocnemius muscle tissues after I/R.

For the first time, this study confirmed that Oxy treatment could attenuate skeletal muscle functional impairment induced by limb I/R by improving mitochondrial function, a phenomenon which was mediated in part by the SIRT1/PGC-1*α* signaling pathway. These results indicated that Oxy treatment might be a promising approach to prevent and treat acute limb I/R injury induced by tourniquet placement. However, some limitations exist in the current study. First, we did not explore the exact mechanism underlying Oxy-induced regulation of acute limb I/R injury induced by tourniquet placement. In addition to the pathways studied, we cannot rule out the possibility that other inflammatory signaling pathways may mediate the protective effect of Oxy treatment. Second, based on the clinically relevant concentrations and concentration used in a previous study [39], we chose a concentration of 0.6 mg/kg of Oxy for intraperitoneal injection in an animal experiment. We did not test the dose-dependent effect of Oxy on I/R injury in the current study. Last, we did not explore the prolonged beneficiary effect of Oxy treatment on skeleton muscle I/R injury. Thus, further investigation is warranted to study the long-term effects of Oxy treatment on the limb experiencing I/R injury and the functions of remote organs following I/R injury induced by tourniquet placement.

## 5. Conclusions

The present study indicated that acute limb I/R induced by tourniquet placement results in morphological and functional impairments in mouse skeletal muscles. Pretreatment with Oxy attenuates acute I/R injury in skeletal muscle through inhibiting TLR4/NF-*κ*B-mediated inflammation and activating SIRT1/PGC-1*α*-dependent mitochondrial biogenesis. Findings from this study provide novel information for developing new therapeutics to reduce skeleton muscle injury induced by tourniquet placement.

## Data Availability

The data used and analyzed during this study are available upon request.

## Abbreviations

Oxy: Oxycodone
SIRTs: Sirtuin family of deacetylases
I/R: Ischemia/reperfusion
PGC-1*α*: Peroxisome proliferator-activated receptor *γ* coactivator-1*α*
TLRs: Toll-like receptors
NF-*κ*B: Nuclear factor kappa B
TNF-*α*: Tumor necrosis factor alpha
IL-1: Interleukin-1
EM: Electron microscope
ELISA: Enzyme-Linked Immunosorbent Assay.

## References

[1] Corrick R. M., Tu H., Zhang D. (2018). Dexamethasone protects against tourniquet-induced acute ischemia-reperfusion injury in mouse hindlimb. *Frontiers in Physiology*.

[2] Blaisdell F. W. (2002). The pathophysiology of skeletal muscle ischemia and the reperfusion syndrome: a review. *Cardiovascular Surgery*.

[3] Smith J. K., Grisham M. B., Granger D. N., Korthuis R. J. (1989). Free radical defense mechanisms and neutrophil infiltration in postischemic skeletal muscle. *The American Journal of Physiology*.

[4] Kue R. C., Temin E. S., Weiner S. G. (2015). Tourniquet use in a civilian emergency medical services setting: a descriptive analysis of the Boston ems experience. *Prehospital Emergency Care*.

[5] Mo Y., Chen S., Yang L. (2017). The effect of transcutaneous electrical acupoint stimulation on inflammatory response in patients undergoing limb ischemia-reperfusion. *Mediators of Inflammation*.

[6] Kaufman R. D., Walts L. F. (1982). Tourniquet-induced hypertension. *British Journal of Anaesthesia*.

[7] Foster A. D., Vicente D., Sexton J. J. (2017). Administration of fty720 during tourniquet-induced limb ischemia reperfusion injury attenuates systemic inflammation. *Mediators of Inflammation*.

[8] Wilson H.-M. P., Welikson R. E., Luo J. (2015). Can cytoprotective cobalt protoporphyrin protect skeletal muscle and muscle-derived stem cells from ischemic injury?. *Clinical Orthopaedics and Related Research*.

[9] Houtkooper R. H., Pirinen E., Auwerx J. (2012). Sirtuins as regulators of metabolism and healthspan. *Nature Reviews. Molecular Cell Biology*.

[10] Rodgers J. T., Lerin C., Haas W., Gygi S. P., Spiegelman B. M., Puigserver P. (2005). Nutrient control of glucose homeostasis through a complex of PGC-1*α* and SIRT1. *Nature*.

[11] Rasbach K. A., Schnellmann R. G. (2008). Isoflavones promote mitochondrial biogenesis. *The Journal of Pharmacology and Experimental Therapeutics*.

[12] O'Neill L. A. (2006). How toll-like receptors signal: what we know and what we don't know. *Current Opinion in Immunology*.

[13] Cen X., Liu S., Cheng K. (2018). The role of toll-like receptor in inflammation and tumor immunity. *Frontiers in Pharmacology*.

[14] Sabroe I., Parker L. C., Dower S. K., Whyte M. K. (2008). The role of tlr activation in inflammation. *The Journal of Pathology*.

[15] Wang S. L., Duan L., Xia B., Liu Z., Wang Y., Wang G. M. (2017). Dexmedetomidine preconditioning plays a neuroprotective role and suppresses TLR4/NF-*κ*B pathways model of cerebral ischemia reperfusion. *Biomedicine & Pharmacotherapy*.

[16] Schmidt-Hansen M., Bennett M. I., Arnold S., Bromham N., Hilgart J. S. (2017). Oxycodone for cancer-related pain. *Cochrane Database of Systematic Reviews*.

[17] Yang P.-P., Yeh G.-C., Huang E. Y.-K., Law P.-Y., Loh H. H., Tao P.-L. (2015). Effects of dextromethorphan and oxycodone on treatment of neuropathic pain in mice. *Journal of Biomedical Science*.

[18] Ye J., Yan H., Xia Z. (2018). Oxycodone ameliorates the inflammatory response induced by lipopolysaccharide in primary microglia. *Journal of Pain Research*.

[19] McCormack M. C., Kwon E., Eberlin K. R. (2008). Development of reproducible histologic injury severity scores: skeletal muscle reperfusion injury. *Surgery*.

[20] Hong Y., Zhang B., Yu L., Duan S. S. (2017). Cell membrane integrity and revascularization: the possible functional mechanism of ischemic preconditioning for skeletal muscle protection against ischemic-reperfusion injury. *Acta Histochemica*.

[21] Gillani S., Cao J., Suzuki T., Hak D. J. (2012). The effect of ischemia reperfusion injury on skeletal muscle. *Injury*.

[22] Albadawi H., Oklu R., Raacke Malley R. E. (2016). Effect of DNase I treatment and neutrophil depletion on acute limb ischemia- reperfusion injury in mice. *Journal of Vascular Surgery*.

[23] Liu Y., Zhou C., Jiang J., Su Q., Ding X. (2017). Blockade of hmgb1 preserves vascular homeostasis and improves blood perfusion in rats of acute limb ischemia/reperfusion. *Microvascular Research*.

[24] Crawford R. S., Hashmi F. F., Jones J. E. (2007). A novel model of acute murine hindlimb ischemia. *American Journal of Physiology. Heart and Circulatory Physiology*.

[25] Wiffen P. J., Wee B., Derry S., Bell R. F., Moore R. A. (2017). Opioids for cancer pain - an overview of cochrane reviews. *Cochrane Database of Systematic Reviews*.

[26] Kharbanda R. K., Peters M., Walton B. (2001). Ischemic preconditioning prevents endothelial injury and systemic neutrophil activation during ischemia-reperfusion in humans in vivo. *Circulation*.

[27] Zhu L., Ye T., Tang Q. (2016). Exercise preconditioning regulates the Toll-like receptor 4/nuclear factor-*κ*B signaling pathway and reduces cerebral ischemia/reperfusion inflammatory injury: a study in rats. *Journal of Stroke and Cerebrovascular Diseases*.

[28] Kim E., Kim H. C., Lee S. (2017). Dexmedetomidine confers neuroprotection against transient global cerebral ischemia/reperfusion injury in rats by inhibiting inflammation through inactivation of the TLR-4/NF-*κ*B pathway. *Neuroscience Letters*.

[29] Hutchinson M. R., Zhang Y., Shridhar M. (2010). Evidence that opioids may have toll-like receptor 4 and md-2 effects. *Brain, Behavior, and Immunity*.

[30] Stevens C. W., Aravind S., Das S., Davis R. L. (2013). Pharmacological characterization of lps and opioid interactions at the toll-like receptor 4. *British Journal of Pharmacology*.

[31] Xie N., Gomes F. P., Deora V. (2017). Activation of *μ*-opioid receptor and Toll-like receptor 4 by plasma from morphine-treated mice. *Brain, Behavior, and Immunity*.

[32] Jiang L., Hu J., He S., Zhang L., Zhang Y. (2016). Spinal neuronal nos signaling contributes to morphine cardioprotection in ischemia reperfusion injury in rats. *The Journal of Pharmacology and Experimental Therapeutics*.

[33] Kuroda Y., Togashi H., Uchida T., Haga K., Yamashita A., Sadahiro M. (2020). Oxidative stress evaluation of skeletal muscle in ischemia-reperfusion injury using enhanced magnetic resonance imaging. *Scientific Reports*.

[34] Ozkok E., Yorulmaz H., Ates G. (2016). Amelioration of energy metabolism by melatonin in skeletal muscle of rats with lps induced endotoxemia. *Physiological Research*.

[35] Huang Y., Chen K., Ren Q. (2018). Dihydromyricetin attenuates dexamethasone-induced muscle atrophy by improving mitochondrial function via the PGC-1*α* pathway. *Cellular Physiology and Biochemistry*.

[36] Theilen N. T., Kunkel G. H., Tyagi S. C. (2017). The role of exercise and tfam in preventing skeletal muscle atrophy. *Journal of Cellular Physiology*.

[37] Lagouge M., Argmann C., Gerhart-Hines Z. (2006). Resveratrol improves mitochondrial function and protects against metabolic disease by activating SIRT1 and PGC-1*α*. *Cell*.

[38] Yang S. R., Wright J., Bauter M., Seweryniak K., Kode A., Rahman I. (2007). Sirtuin regulates cigarette smoke-induced proinflammatory mediator release via rela/p65 nf-kappab in macrophages in vitro and in rat lungs in vivo: implications for chronic inflammation and aging. *American Journal of Physiology. Lung Cellular and Molecular Physiology*.

[39] Thorn D. A., Siemian J. N., Zhang Y., Li J. X. (2015). Anti-hyperalgesic effects of imidazoline i2 receptor ligands in a rat model of inflammatory pain: interactions with oxycodone. *Psychopharmacology*.

